# Transcription Factor Eb Is Required for Macropinocytosis-Mediated Growth Recovery of Nutrient-Deprived Kras-Mutant Cells

**DOI:** 10.3390/nu10111638

**Published:** 2018-11-02

**Authors:** Seokmin Jeong, Jun-Kyu Byun, Sung Jin Cho, Jungwook Chin, In-Kyu Lee, Yeon-Kyung Choi, Keun-Gyu Park

**Affiliations:** 1 Department of Biomedical Science, Kyungpook National University, Daegu 41566, Korea; wjdtjrals03@nate.com; 2 Research Institute of Pharmaceutical Sciences, College of Pharmacy, Kyungpook National University, Daegu 41566, Korea; byun0504@hotmail.com; 3 New Drug Development Center, Daegu-Gyeongbuk Medical Innovation Foundation, Daegu 41061, Korea; sjcho@dgmif.re.kr (S.J.C.); jwchin@dgmif.re.kr (J.C.); 4 Department of Internal Medicine, School of Medicine, Kyungpook National University, Kyungpook National University Hospital, Daegu 41944, Korea; leei@knu.ac.kr

**Keywords:** KRAS, macropinocytosis, TFEB, lysosome

## Abstract

Macropinocytosis is a regulated form of endocytosis that mediates the nonselective uptake of nutrients to support growth under nutrient-deprived conditions. KRAS-mutant cancer cells upregulate macropinocytosis to import extracellular proteins, which subsequently undergo proteolytic degradation in the lysosome. Although transcription factor EB (TFEB) is a master regulator of lysosomal biogenesis and function, its role in the degradation of extracellular protein from macropinocytosis in KRAS-mutant cells has not previously been elucidated. In this study, we investigated the role of TFEB in the recovery of macropinocytosis-mediated mTORC1 activity and cell growth under nutrient depletion. Mouse embryonic fibroblasts (MEFs) expressing *Kras*^G12D^ and KRAS-mutant human cancer cells took up markedly higher levels of tetramethylrhodamine (TMR)-dextran than the corresponding wild-type cells. siRNA-mediated inhibition of TFEB did not influence extracellular TMR-dextran uptake, but significantly attenuated lysosomal degradation of extracellular protein. Bovine serum albumin (BSA) treatment restored p-S6K levels and cell proliferation suppressed by leucine deprivation, and these effects were blocked by si*TFEB*. Collectively, our results show that TFEB plays a role in macropinocytosis-mediated KRAS-mutant cell growth under nutrient deprivation by promoting lysosomal degradation of extracellular proteins.

## 1. Introduction

Macropinocytosis is a fluid-phase endocytic process whereby extracellular fluid and its content are internalized into cells through large uncoated vacuoles called [1]. Oncogenic proteins of the RAS family of small guanosine triphosphatases (GTPases) upregulate membrane ruffling and macropinocytosis, allowing cancer cells harboring RAS mutations to use extracellular protein obtained through macropinocytosis to maintain their rapid growth [2]. In addition to oncogenic mutations and growth factors, membrane-bound phospholipids, in particular phosphoinositides and other protein kinases such as p21-activated kinase 1 (Pak1) and protein kinase C, have been implicated in the process of macropinocytosis [3,4]. Inhibition of the mechanistic target of rapamycin complex 1 (mTORC1) promotes macropinocytosis, which is required for cell growth under amino acid starved condition [5]. Moreover, lysosomal delivery of fluid-phase cargo, including protein, and lysosomal degradation are both important for maintaining cell growth under amino acid starvation [6].

Transcription factor EB (TFEB), a member of the MiTF/TFE family, is a master regulator of multiple cellular processes, including lysosomal biogenesis and autophagy [7]. TFEB regulates the expression of a large number of target genes, thereby orchestrating cellular degradative pathways and intracellular clearance in response to environmental cues [8]. In addition, TFEB has attracted attention because of its ability to promote cell proliferation through transcriptional regulation of genes involved in lysosomal–autophagic pathways [9,10]. Indeed, altered TFEB expression is associated with initiation and progression of multiple types of cancer, including lung and pancreatic cancers [11,12,13]. However, it remains unclear whether TFEB plays a role in macropinocytosis-mediated cell growth upon nutrient starvation. Hence, we investigated the role of TFEB in the macropinocytosis-mediated recovery mTORC1 activity and cell growth, both of which are suppressed by nutrient deprivation.

## 2. Materials and Methods

### 2.1. Cell Culture

*Kras*^G12D^ and *Kras*^WT^ mouse embryonic fibroblasts (MEFs) (a gift from Dr. Craig B. Thompson, Memorial Sloan Kettering Cancer Center, New York, NY, USA) were obtained from Cre recombinase-induced SV40 large T-immortalized Lox-Stop-Lox-K-Ras^G12D^ MEF [14]. MEFs and the pancreatic cancer cell line MIA PaCa-2 were cultured in DMEM medium (WELGENE, Gyeongsan-si, Korea) containing 10% fetal bovine serum and 1% penicillin/streptomycin. Another pancreatic cancer cell line, BXPC-3, and lung cancer cell lines A549 and H522 were cultured in RPMI 1640 medium (WELGENE) containing 10% fetal bovine serum and 1% penicillin/streptomycin. For leucine deprivation, cells were maintained in leucine-free DMEM or RPMI 1640 containing 10% dialyzed fetal bovine serum (FBS). MIA PaCa-2, A549, and H522 were obtained from Korean Cell Line Bank (Seoul, Korea) and BXPC-3 was obtained from the American Type Culture Collection (Manassas, VA, USA).

### 2.2. Fluorescence Microscopy

Cell were treated with 1 mg/mL TMR-dextran 70 kDa (Invitrogen, Carlsbad, CA, USA) in leucine-free medium for 3 h, or with 0.5 mg/mL DQ-BSA (Invitrogen) in leucine-free medium for 6 h. Cells were washed three times with ice-cold phosphate-buffered saline (PBS), and then fixed with 3.7% formaldehyde. After fixation, cells were mounted in mounting solution containing DAPI (Vector Laboratories, Burlingame, CA, USA). For imaging of DQ-BSA fluorescence and lysosome, cells were treated with 0.5 mg/mL DQ-BSA (Invitrogen) for 3 h and 50 nM Lysotracker Red (Invitrogen) for 1 h prior to analysis in leucine-free medium. Nuclei were stained using NucBlueTM Live ReadyProbesTM Reagent (Invitrogen). Images were analyzed using the ‘Analyze Particle’ tool in Image J (a Java-based image processing program).

### 2.3. siRNA Knockdown

For gene silencing, cells were transfected with scrambled siRNA, si*KRAS*, and si*TFEB* (Bioneer, Daejeon, Korea) using Lipofectamine RNAiMAX reagent (Invitrogen).

### 2.4. Western Blot Analysis

Cells were lysed in lysis buffer containing 20 mM Tris-HCl (pH 7.4), 5 mM EDTA (pH 8.0), 10 mM Na_4_P_2_O_7_, 100 mM NaF, 2 mM Na_3_VO_4_, 1% NP-40, and protease and phosphatase inhibitors. Proteins from cellular lysates were resolved on a 10% sodium dodecyl sulfate polyacrylamide gel electrophoresis (SDS-PAGE) gel and transferred to a polyvinylidene difluoride (PVDF) membrane. Membranes were blocked with 5% skimmed milk and incubated overnight at 4 °C with primary antibodies at the indicated dilutions: antibodies against p70 S6 kinase, phospho-p70 S6 Kinase (Thr389), and TFEB (1:1000) were obtained from Cell Signaling Technology (Beverly, MA, USA), and antibody against β-actin (1:5000) was obtained from Santa Cruz Biotechnology (Dallas, TX, USA). After incubation with primary antibodies, membranes were washed three times with Tris-buffered saline containing 0.1% Tween 20 (TBST), and then incubated with horseradish peroxidase (HPR)-conjugated rabbit secondary antibody (Cell Signaling Technology). HRP was detected using the WEST-Queen^TM^ Western Blot Detection Kit (iNtRON Biotechnology, Seongnam, Korea).

### 2.5. Cell Proliferation

Cells were transfected with scrambled siRNA or si*TFEB* for 24 h and then maintained in leucine-free medium with or without 3% BSA (Sigma, St. Louis, MO, USA) and EIPA (Sigma) for 72 h. Cell proliferation was measured using a CCK-8 assay (Dojindo Molecular Technologies, Rockville, MD, USA).

### 2.6. Statistical Analysis

All values are presented as means ± SEM. Statistical analysis was performed using an unpaired *t*-test. *p* < 0.05 was considered statistically significant.

## 3. Results

### 3.1. KRAS-Mutant Cells Exhibit Higher Levels of Macropinocytosis Than Kras Wild-Type Cells

**Figure 1:**
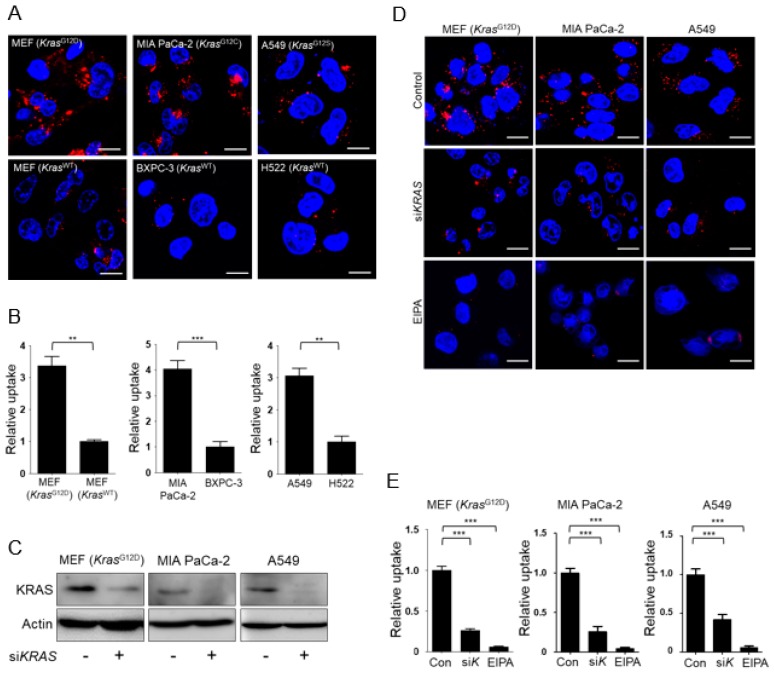
KRAS-mutant cells exhibit elevated levels of macropinocytosis. (**A**) Uptake of extracellular tetramethylrhodamine (TMR)-dextran as a marker of macropinosomes (red) in KRAS-mutant (*Kras*^G12D^ MEFs, MIA PaCa-2, and A549) and wild-type cell lines (wild-type MEFs, BXPC-3, and H522). Cells were maintained in leucine-free medium for 24 h, followed by treatment with TMR-dextran for 3 h. (**B**) Quantification of macropinosomes (red) in cells shown in (**A**). Data are expressed as means ± SEM from three independent experiments. (**C**) Western blot analysis showing the knockdown efficiency of si*KRAS*. Cells were transfected with scrambled siRNA or si*KRAS* for 24 h and then maintained in leucine-free medium for 24 h. (**D**) Uptake of extracellular TMR-dextran as a marker of macropinosomes (red) in KRAS-mutant cells. Cells were transfected with scrambled siRNA (Control, Con) or si*KRAS* (si*K*) for 24 h, maintained in leucine-free medium for 24 h, and then with TMR-dextran in the presence or absence of 30 μM EIPA for 3 h. (**E**) Quantification of macropinosomes (red) in cells shown in (**D**). Data were normalized with control siRNA-transfected cells and expressed as means ± SEM of five images with at least 20 cells per treatment group. ** *p* < 0.01; *** *p* < 0.001. Scale bar, 2 μm.

First, we compared fluid-phase uptake in *Kras*^G12D^ MEFs and KRAS-mutant human pancreatic (MIA PaCa-2) and lung (A549) cancer cells with the corresponding KRAS wild-type cells (MEFs, BXPC-3 human pancreatic cancer cells, and H522 human lung cancer cells). Macropinosomes were detected using tetramethylrhodamine-labeled high-molecular-mass dextran (TMR-dextran), an established marker of macropinocytosis, on the basis of cells’ ability to internalize extracellular fluid and its contents [15]. Consistent with previous results, TMR-dextran uptake was higher in Kras^G12D^ MEFs and KRAS-mutant human cancer cells than in the respective wild-type controls (Figure 1A,B). Next we evaluated whether knockdown of KRAS attenuated macropinocytosis. Successful knockdown of KRAS was confirmed by Western blot analysis (Figure 1C). Increased rates of macropinocytosis in *Kras*^G12D^ MEFs and KRAS-mutant human cancer cells were significantly attenuated by knockdown of KRAS or treatment with a pharmacological inhibitor of macropinocytosis, 5-(*N*-ethyl-*N*-isopropyl)-amiloride (EIPA) (Figure 1D,E).

### 3.2. TFEB Promotes Lysosomal Degradation of Extracellular Protein without Affecting Macropinocytotic Uptake

**Figure 2:**
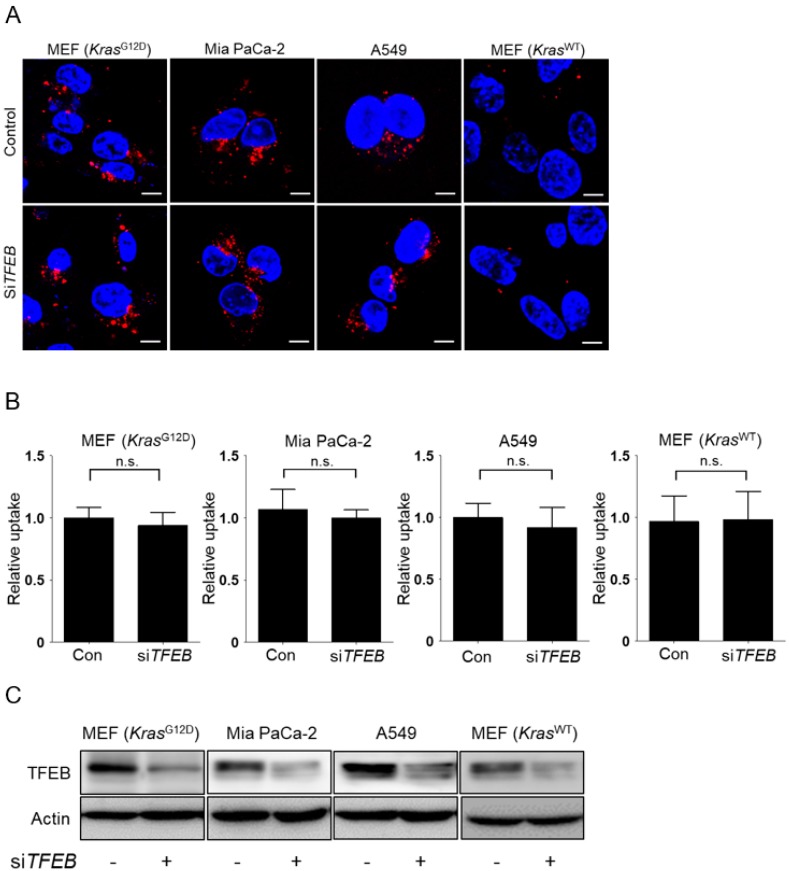
Knockdown of transcription factor EB (TFEB) does not influence uptake via macropinocytosis in KRAS-mutant cells. (**A**) Uptake of extracellular TMR-dextran as a marker of macropinosomes (red) in KRAS-mutant cells. Cells were transfected with scrambled siRNA (Control, Con) or si*TFEB* for 24 h, maintained in leucine-free medium for 24 h, and then treated with TMR-dextran for 3 h. (**B**) Quantification of macropinosomes in cells shown in (**A**). (**C**) Western blot analysis showing the knockdown efficiency of si*TFEB*. Cells were transfected with scrambled siRNA or si*TFEB* for 24 h, and then maintained leucine-free medium for 24 h. Data were normalized with control siRNA-transfected cells and expressed as means ± SEM of five images with at least 20 cells per treatment group. n.s., not significant. Scale bar, 2 μm.

**Figure 3:**
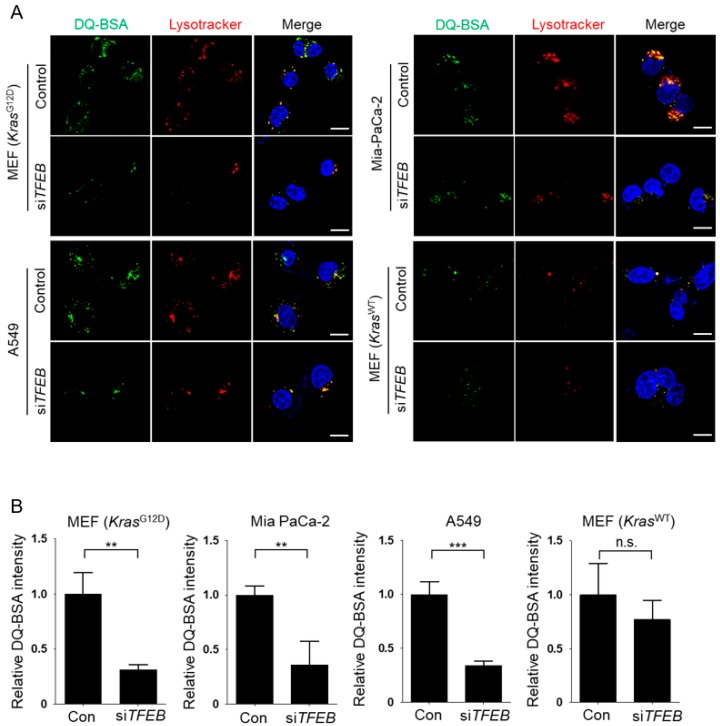
Knockdown of transcription factor EB (TFEB) decreases lysosomal proteolysis of extracellular albumin in KRAS-mutant cells. (**A**) Intracellular degradation of BSA (green) in KRAS-mutant cells and *Kras*^WT^ MEFs. Cells were transfected with scrambled siRNA (Control, Con) or si*TFEB* for 24 h, maintained in leucine-free medium for 24 h, and then treated with DQ-BSA for 3 h and Lyso Tracker (red) for 1 h. (**B**) Quantification of DQ-BSA fluorescence in cells shown in (**A**). Data were normalized with control siRNA-transfected cells and expressed as means ±SEM of five images with at least 20 cells per treatment group. n.s., not significant; ** *p* < 0.01; *** *p* < 0.001. Scale bar, 1 μm.

Next, we investigated whether TFEB contributes to the macropinocytic pathway in KRAS-mutant cells. In KRAS-mutant cells, siRNA-mediated knockdown of *TFEB* did not affect macropinocytotic uptake, as measured by TMR-dextran incorporation under leucine-depleted conditions (Figure 2A,B). By contrast, the *Kras*^WT^ MEFs negative control did not show altered macropinocytic uptake upon leucine depletion, irrespective of TFEB expression (Figure 2A,B). Successful knockdown of TFEB was confirmed by Western blot analysis (Figure 2C). To assay lysosomal proteolytic activity, KRAS-mutant cells were incubated with boron-dipyrromethene (BODIPY)-conjugated bovine serum albumin (DQ-BSA), which is taken up by macropinocytosis and fluoresces after lysosomal degradation, and with Lysotracker to label lysosomes. TFEB knockdown significantly decreased Lysotracker labeling and the number of DQ-BSA fluorescent puncta in leucine-starved KRAS mutant cells, but not in *Kras*^WT^ MEFs (Figure 3A,B), suggesting that TFEB plays a critical role in lysosomal biogenesis and lysosomal proteolysis of extracellular albumin taken up by macropinocytosis in KRAS-mutant cells.

### 3.3. TFEB Contributes to Macropinocytosis-Mediated Recovery of mTORC1 Activity and Cell Proliferation in Leucine-Deprived KRAS-Mutant Cells

**Figure 4:**
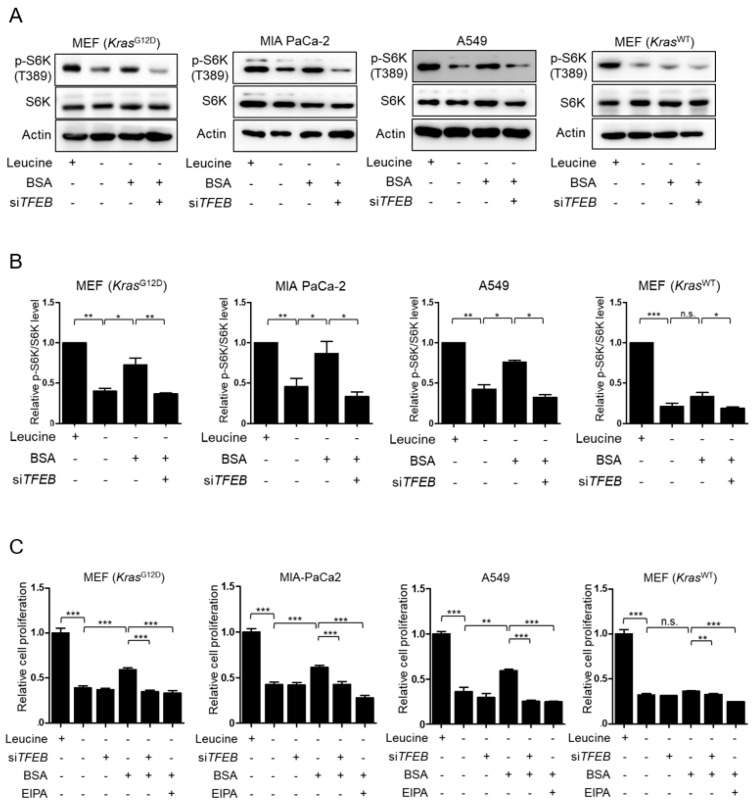
Transcription factor EB (TFEB) plays a role in macropinocytosis-mediated recovery of mTORC1 activity and cell growth in KRAS-mutant cells. (**A**) Representative Western blot showing mTORC1 activation in KRAS-mutant cells. Cells were transfected with scrambled siRNA or si*TFEB* for 24 h, and then maintained in leucine-free medium for 24 h with or without 3% BSA. (**B**) Quantitative densitometric data of phospho/total S6K abundance shown in (**A**). The intensity of each band was measured using ImageJ software. (**C**) Cells were transfected with scrambled siRNA or si*TFEB* for 24 h, and then maintained in leucine-free medium with or without 3% BSA or 10 μM EIPA for 72 h. Cell proliferation was measured using a CCK-8 assay. Data were normalized with control siRNA-transfected cells in leucine-replete media and expressed as means ±SEM of three independent experiments. n.s., not significant. * *p* <0.05; ** *p* < 0.01; *** *p* < 0.001.

Because mTORC1 activity is suppressed under amino acid starvation, we asked whether TFEB-mediated lysosomal degradation of extracellular protein could restore suppressed mTORC1 activity in leucine-depleted cells. As shown in Figure 4A,B, treatment of *Kras*^G12D^ MEFs, MIA PaCa-2, and A549 cells with BSA restored mTORC1 activity suppressed by leucine deprivation, as measured by phosphorylation of S6K (T389). However, this recovery of mTORC1 activity was prevented when TFEB was silenced, indicating that TFEB restored mTORC1 activity in response to BSA stimulation through macropinocytosis. By contrast, treatment of *Kras*^WT^ MEFs with BSA did not influence mTORC1 activity in the leucine deprivation condition; neither was affected by silencing of TFEB. Consistent with the changes in mTORC1 activity, treatment with BSA exhibited recovery of cell proliferation, which was also suppressed by leucine starvation in *Kras*^G12D^ MEFs, MIA PaCa-2 and A549 cells, but not in *Kras*^WT^ MEFs (Figure 4C). In addition, knockdown of TFEB abolished BSA-induced recovery of cell growth under leucine deprivation in KRAS-mutant cells (Figure 4C). Macropinocytosis-dependent recovery of cell growth was confirmed by EIPA treatment (Figure 4C). Although si*TFEB* and EIPA reduced BSA-treated *Kras*^WT^ MEF proliferation in the leucine-starved condition, their effect on proliferation in these cells was minimal compared with their effect on proliferation in KRAS-mutant cells. Collectively, these data demonstrated that TFEB contributes to sustained cell proliferation by promoting lysosomal catabolism of extracellular albumin taken up by macropinocytosis.

## 4. Discussion

In this study, we demonstrated that TFEB plays a pivotal role in acquisition of nutrients required for growth in KRAS-mutant cancer cells under nutrient-depleted conditions. Our results showed that TFEB does not affect macropinocytic uptake, but is responsible for lysosomal catabolism of internalized protein and maintenance of intracellular amino acid availability in KRAS-mutant cancer cells. Therefore, inhibition of TFEB could prevent metabolic adaptation of cancer cells to energy starvation, and thus represents a promising strategy for the treatment of cancers harboring KRAS mutations. 

Cancer cells can maintain metabolic homeostasis to sustain growth and survival under nutrient limitation by activating stress responses [16,17]. Several lines of evidence show that macropinocytosis plays crucial roles in maintaining nutrient supply under nutrient-deficient conditions [2,5]. Macropinocytosis occurs at basal rate in some cell types, including macrophages and dendritic cells, to allow uptake of antigen for subsequent presentation [18]. By contrast, in cancer cells, macropinocytosis is dramatically induced by receptor kinases or oncogenic signaling by RAS [19,20]. RAS stimulates actin-driven membrane ruffling and macropinosome formation [1]. Consistent with this, our study also showed that KRAS-mutant cells take up higher levels of TMR-dextran than *KRAS*^WT^ cells, and can therefore sustain rapid proliferation in the absence of nutrients.

In addition to oncogenic signaling, several factors are necessary for coordinately regulating macropinocytosis [1]. Although mTORC1 has growth-promoting activity when extracellular nutrients are plentiful, suppression of mTORC1 under nutrient-depleted conditions protects proliferating cells from energetic stress by maintaining energy and redox balance [17]. Furthermore, lysosomal degradation in response to mTORC1 inhibition promotes cell proliferation under amino acid starvation [5]. In this study, we found that when KRAS-mutant cells were placed in medium containing DQ-BSA, mTORC1 activity and cellular proliferation suppressed by leucine deprivation were significantly recovered. Under nutrient-limited conditions, suppression of mTORC1 activity or elevation of calcineurin activity induces dephosphorylation of TFEB, leading to its nuclear translocation, and subsequently to upregulation of lysosomal biogenesis [21,22]. On the other hand, recent study showed that TFEB influences mTORC1 activity, consisting of a feedback loop through transcriptional regulation of RagD expression. TFEB or TFE3 overexpression during starvation induces RagD expression and assembly of inactive Rags heterodimers on the lysosome, which can boost mTORC1 activation in response to nutrients [23]. Accordingly, TFEB can compensate for nutrient shortage by promoting generation of metabolic intermediates required for cancer growth under nutrient-depleted conditions. Indeed, we found that TFEB was responsible for recovery of cellular proliferation mediated by breakdown of extracellular albumin scavenged through macropinocytosis, but did not affect cellular uptake of fluorescently labeled dextran or albumin. Thus, our study suggests that TFEB maintains intracellular amino acid availability without affecting bulk internalization of extracellular proteins, allowing cancer cells to proliferate even under nutrient deprivation.

In summary, this study shows that TFEB is an important metabolic regulator in macropinocytosis-mediated KRAS-mutant cell growth under nutrient-deprived conditions. TFEB initiates anabolic pathways and cellular adaptation to metabolic stress by promoting lysosomal degradation of extracellular proteins. TFEB-mediated lysosomal catabolism of macropinocytic proteins confers a growth advantage on KRAS-mutant cells. Future studies should explore how the function of TFEB could be therapeutically targeted.
